# Does scientific effort reflect global need? A review of infectious disease publications over 100 years

**DOI:** 10.1017/S0950268818003552

**Published:** 2019-03-01

**Authors:** D. T. S. Hayman, M. G. Baker

**Affiliations:** 1 ^m^EpiLab, Infectious Diseases Research Centre, Massey University, Palmerston North 4442, New Zealand; 2 University of Otago, Wellington, New Zealand

**Keywords:** DALY, emerging infectious diseases, global burden of disease, health equity, neglected tropical diseases

## Abstract

In a rational world, scientific effort would reflect society's needs. We tested this hypothesis using the area of infectious diseases, where the research response to emerging threats has obvious potential to save lives through informing interventions such as vaccination and prevention policies. Pathogens continue to evolve, emerge and re-emerge and infectious diseases that were once common become less so or their global distribution changes. A question remains as to whether scientific endeavours can adapt. Here, we identified papers on infectious diseases published in the four highest ranking, health-related journals over the 118 years from 1900. Focussing on outbreak-related and burden of disease-related metrics over the two time periods, 1990 to 2017 and 1900 to 2017, our analyses suggest that there is little underrepresentation of important infectious diseases among top ranked journals. Encouragingly our results suggest the scientific process is largely self-correcting.

## Introduction

In a rational research environment, science effort would reflect need. We tested this hypothesis using the area of infectious diseases, where a rapid and proportionate research response to emerging threats has obvious potential to identify life-saving interventions such as new diagnostic tests, vaccines and guidance for prevention policies. Pathogens continue to evolve, emerge and re-emerge so it would be reassuring to know if research was similarly adaptive.

### Dynamic nature of infectious disease emergence

The world's current greatest infectious causes of mortality are human immunodeficiency virus (HIV) and tuberculosis (TB). HIV was first reported in 1981 and the HIV viruses are classic examples of zoonotic disease events that emerge as outbreaks with some viruses becoming global pandemic infections [1, 2]. HIV, and outbreaks such as the West African Ebola virus outbreak, have led to calls for increasing studies on emerging infectious diseases [3].

However, frequently fatal infectious diseases have been with us longer. TB infects approximately one in four people, killing approximately 1.7 million a year [4]. The earliest discovered TB cases in people are found in skeletal remains from 4000BCE and it may have emerged with Neolithic people [5]. TB is often considered the quintessential re-emerging disease, particularly during the 1980s as case numbers increased across the globe [6].

TB, HIV and malaria are the focus for control initiatives, including millennium development goals and Gates Foundation programmes that aim to reduce their global burden, because of their high morbidity and mortality. Beyond these high-profile diseases sit a range of other infectious diseases, some apparently neglected because of their distribution across less affluent tropical regions, and some common, but causing less mortality [7].

### Infectious disease importance and journal publications

We may expect a proportionate research effort to the impact of infectious diseases and for this balance to be reflected in the scientific literature. However, academics face a number of challenges achieving this equilibrium. Stochastic article acceptance into the top-ranking journals, along with putatively aberrant reward systems and mixed funding opportunities, may lead to some areas of research being neglected among highly ranked scientific journals. As scientists interested in emerging and neglected infectious diseases, health inequalities and policy, we hypothesised that some globally important infectious diseases would be underrepresented in the highest-ranking scientific journals. We test this hypothesis with statistical analyses of publications citing infectious agents in the four highest-ranking and longest running medico-scientific journals.

## Methods

### Search strategy and selection criteria

To test our hypothesis we classified importance in two broad ways: outbreak frequency and disease burden. For outbreak frequency data we used the most frequent outbreak-related infectious diseases from 1980 to 2010 [8]. For disease burden we used mortality (deaths), years of life lost (YLL) from premature death, and disability-adjusted life years (DALY, which combine YLL and years lost from disabilities), from 2006 to 2016 reported in the global burden of disease (GBD) studies [9, 10].

**Fig. 1.**
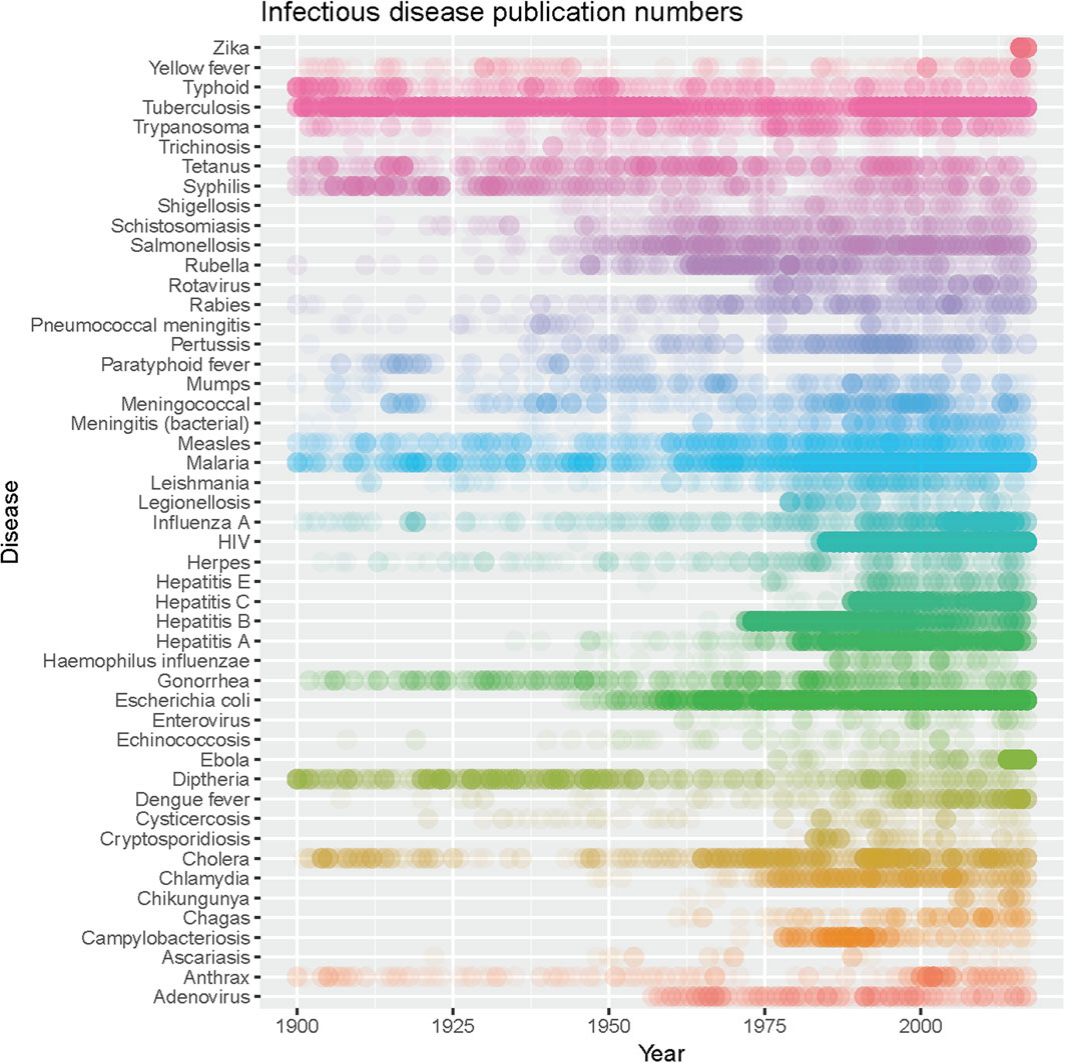
Incidence of published studies of infectious diseases used in this study in four major journals from 1900 to 2017. Colour density represents the number of publications each year for all journals. Incidence by journal is included in the supplementary information.

From these two sources we identified a list of key infectious diseases (including organisms or syndromes if caused by a range of infectious agents). We identified the four highest ranking, cited publications by Google scholar (Nature, Science, New England Journal of Medicine and The Lancet), and searched for those infectious diseases using Web of Science (Supplementary information). Data were extracted from Web of Science using the search terms in the online Excel file for infectious diseases and the journal names. These journals also had the advantage of having continuous publication records extending back for more than a century, providing additional insights into historic patterns of research attention, and we used all publications in these journals up to the search date (Fig. 1), though used subsets of the data for specific analyses (see below). One final search was specifically performed for smallpox (see below).

### Analyses

Boxplot.stats in R was used to identify the outliers, either in the number of publications for each disease for the 1980–2010 outbreak data or from the residuals from a generalised linear model with Poisson error distribution using the glm function in R, where the number of publications was the outcome and the burden metric the predictor. Thus, outliers were identified as those which were outside 1.5 times the interquartile range. Overrepresented infectious diseases were outside the upper quartile and underrepresented below the lower quartile.

For smallpox, annual publication rates and confidence intervals were estimated using the mean and Poisson confidence intervals (i.e. 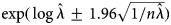), where 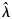 is the mean and *n* is the number of years. Statistical significance between publication rates by journal was determined using 95% confidence intervals (CI). All data manipulations and figures were plotted in ggplot2 [11] or base R [12].

### Publication trends

For 26 outbreak-related infectious diseases, there were 19 685 publications from 1900 to October 2017. Quartile statistics for the 1990–2010 period and for all years suggested none were under-represented (Supplementary information). TB, malaria and *Escherichia coli* were overrepresented (i.e. outliers with greater numbers of publications) for the entire time series, with the addition of hepatitis B for the 1990–2010 period. We did not distinguish the purpose of publications, but of the 1803 *E. coli* publications, we believe many are reports of it as a model research organism, likely explaining its overrepresentation. No changes were seen when splitting the data into zoonotic (animal-origin) and human only (Supplementary information).

**Fig. 2.**
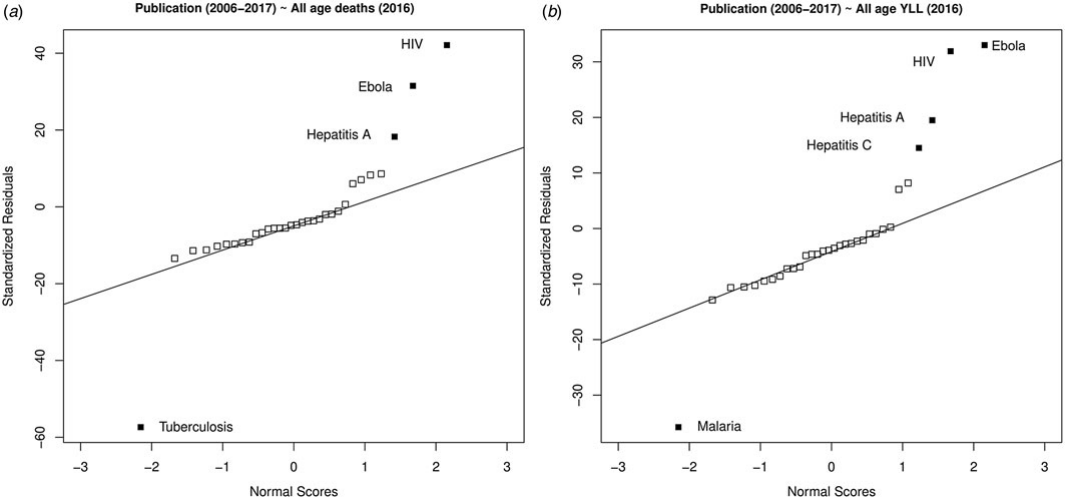
Over or underrepresented infectious diseases in the published literature in four major journals according to GBDs study (2016) [9, 10] by (a) All age deaths and (b) All age years of life lost (YLL). Analyses of publication from 2006 to 2017 period are shown with the residuals of Poisson regression models and their outlier significance (*α* = 0.05, solid filled points) shown. Those filled points above the line represent infections overrepresented in the literature, those filled points below the line are underrepresented.

For 49 burden of disease-related infectious diseases, there were 37 140 publications from 1900 to 2017 (Supplementary information). To allow comparison with burden of disease, we focused on the more recent period 2006–2017. Poisson generalised linear regression model residuals for this period identified outliers (Fig. 2). Only malaria and TB were identified as underrepresented on DALY, YLL and mortality models. However, TB was only underrepresented for the mortality and malaria for both DALY and YLL measures, which are related, and both infectious diseases were very highly studied.

HIV was consistently overrepresented. Hepatitis A was significantly overrepresented in the 2006–2017 publications, and a non-significant outlier in the overall DALY analysis. Ebola virus disease (EVD) and hepatitis C were the only other significantly overrepresented disease, and hepatitis C not a significant outlier in the mortality analysis. We believe the West African EVD was not only a significant globally important outbreak but coincided with advances in data generation through near real-time, whole genome sequencing, increasing the likelihood of publication in high ranking public health and multi-disciplinary journals.

When we compared the percentage change in DALY, YLL and mortality between 2006 and 2016 no infectious disease was underrepresented in publications from the 2006 to 2017 period. However, TB, malaria and HIV were overrepresented in all estimates and hepatitis B in the mortality model.

**Fig. 3.**
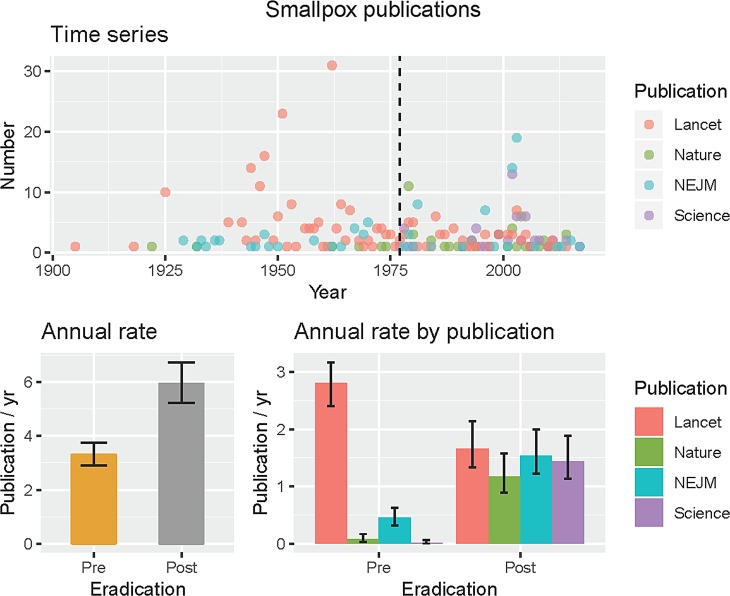
Smallpox publications in four major journals. The last case was reported in 1977 (black dashed line, time series), and was used to define pre- and post-eradication periods as the burden of disease was then zero. Mean rates with 95% CIs pre- and post-eradication are shown for all four journals together and by journal.

Finally, in the long-term datasets (Fig. 1), we can see some interesting changes in publications over the 118 years of publications. Publications relating to TB and malaria remain dominant in the literature, whereas there are declines in diphtheria and other vaccine-preventable diseases. Out of interest, we performed the same search for smallpox, because this is the one human pathogen globally eradicated, so now has zero burden of disease (Supplementary information). We discovered an increased annual rate of publication from pre-eradication (⩽1977, 3.3 mean, 2.9–3.8 95% CI) to post-eradication (>1977, 6.0 mean, 5.2–6.7 95% CI) in these journals, using the last case in 1977 rather than formal eradication in 1980 as a breakpoint. However, the rates differed significantly by journal, in particular The Lancet published at a greater annual rate prior to eradication, whereas the other three journals increased their publication rates after smallpox eradication (Fig. 3). We suspect this sustained interested was because of concerns regarding smallpox reintroduction [13, 14].

### Policy implications

What scientists’ choose to research is crucial to the advancement of society. That there is little evidence of underrepresentation of important infectious diseases among these top ranked journals is encouraging. Science has been assumed to be self-correcting, and our analysis of the publication record from two periods, 1990–2017 and 1900–2017, in relation to burden of disease measures, suggests self-correction may happen (Figs 1 and 2, Supplementary information).

Our analyses could provide a baseline for future studies. We believe the long-term approach of our study allows us to observe the processes of self-correction and ‘regression to the mean’ over time. We focused on specific infectious diseases, but clearly non-communicable diseases are now the leading causes of morbidity and mortality globally and comparable analyses could be done to see whether publication trends follow disease burden in these areas.

Our analysis has several important limitations. Focusing on specific pathogens means that some important infectious disease problems, such as antimicrobial resistance, are not specifically included. Number of publications in an area is an imperfect measure of research effort. For example, it will not measure whether sufficient research is being carried out in low and middle-income countries. These limitations could be addressed in future studies. Refinements through improved search algorithms would help differentiate the focus of >37 000 publications (e.g. *E. coli* with 1803 publications). Similar improvements may help differentiate among syndromes, something we avoided because we could not differentiate aetiologies. Our use of recent GBDs will almost certainly have missed important changes in this with time. Timing of research efforts could also be examined in a more granular way. For example, whether the lag from identification of HIV as an emerging infectious disease until maximum research effort occurred was slower than is seen for more recent emerging threats such as EVD, and whether these delays matter.

Analyses to determine the relationships between funding, publication, policy and disease burden are required to further improve health outcomes. The coverage of science research provided by the four high impact journals used here, however, provides insights into research effort for a remarkable long period of modern science endeavour from 1900 to 2017 (Fig. 1 and Supplementary information). Whilst some idiosyncrasies exist, as highlighted by our analysis of smallpox, we were encouraged that like recent analyses of bias in science [15], we found our initial hypotheses incorrect and the scientific process appears to largely be self-correcting.

## Supplementary material

For supplementary material accompanying this paper visit https://doi.org/10.1017/S0950268818003552. click here to view supplementary material
